# The Effect of a Peanut-Enriched Weight Loss Diet Compared to a Low-Fat Weight Loss Diet on Body Weight, Blood Pressure, and Glycemic Control: A Randomized Controlled Trial

**DOI:** 10.3390/nu14142986

**Published:** 2022-07-21

**Authors:** Kristina S. Petersen, Jess Murphy, Jane Whitbread, Peter M. Clifton, Jennifer B. Keogh

**Affiliations:** 1 Department of Nutritional Sciences, Texas Tech University, Lubbock, TX 79409, USA; 2 Clinical and Health Sciences, University of South Australia, Adelaide, SA 5000, Australia; jess.murphy@unisa.edu.au (J.M.); jane.whitbread@unisa.edu.au (J.W.); peter.clifton@unisa.edu.au (P.M.C.); jennifer.keogh@unisa.edu.au (J.B.K.)

**Keywords:** weight loss, peanuts, overweight, obesity, prediabetes

## Abstract

The objective of this study was to examine the effect of consuming 35 g of peanuts prior to two main meals per day as part of a weight loss diet, compared to a traditional low-fat weight loss diet, on body weight, markers of glycemic control, and blood pressure in adults at risk of type 2 diabetes over 6 months. A two-arm randomized controlled trial was conducted. Adults (age > 18 years) with a BMI of >26 kg/m^2^ at risk of type 2 diabetes were randomized to the peanut group or the traditional low-fat-diet group (control). The peanut group was advised to consume 35 g of lightly salted dry-roasted peanuts prior to two main meals per day. Participants in the control group were given education to follow a low-fat diet. Both groups had dietetic counseling to restrict energy intake (women: <5500 kJ/1300 kcal/d; men: <7000 kJ/1700 kcal/d). Outcome assessment occurred at baseline, 3 months, and 6 months. In total, 107 participants were randomized (65% female; mean age 58 ± 14 years, BMI 33 ± 5.4 kg/m^2^, waist circumference 109 ± 13 cm, AUSDRISK score 15 ± 5 points), and 76 participants completed the study. No between-group difference in body weight (primary outcome) was observed at 6 months (mean difference, −0.12 kg; 95% CI, −2.42, 2.18; *p* = 0.92). The mean weight loss at 6 months was 6.7 ± 5.1 kg in the cohort (visit *p* < 0.001). HbA1c, fasting glucose, fasting insulin, 2-h glucose, and HOMA-IR were not different between the groups. Systolic blood pressure was reduced to a greater extent in the peanut group vs. the control group at 6 months (−5.33 mmHg; 95% CI, −9.23, −1.43; *p* = 0.008). Intake of 35 g of peanuts prior to two main meals per day, in the context of an energy-restricted diet, resulted in weight loss comparable to a traditional low-fat weight loss diet without preloads. Greater systolic blood pressure reductions were observed with peanut intake, which may lower cardiovascular disease risk.

## 1. Introduction

Overweight and obesity continues to be an issue of global public health significance. In the United States, approximately 74% of adults aged >20 years have overweight or obesity [1]. Similarly, in Australia, 67% of adults had overweight or obesity in 2017/2018, an increase from 63.4% in 2014/2015 [2]. In young Australian adults (18–24 years), overweight and obesity has increased from 38.9% in 2014/2015 to 46.0% in 2017/2018. Overweight and obesity significantly increase the risk of type 2 diabetes and cardiovascular disease (CVD) [3,4]. Dietary approaches that assist adults with overweight and obesity to achieve sustained weight loss are critical for type 2 diabetes and CVD risk reduction.

The first-line intervention for treatment of overweight and obesity is an energy-restricted diet [5]; however, many barriers to adopting and sustaining an energy-restricted diet exist. A key challenge is feelings of hunger because of the lower satiety value of many weight loss diets. Higher-protein diets have greater satiety value and are one dietary approach recommended for weight loss [5]. Another strategy that may promote satiety and assist with lowering energy intake is consuming a preload prior to main meals. A recent randomized trial showed that prescription of an energy-restricted diet (−500 kcal/d) and intake of a high-protein, fiber-based shake (17 g protein, 6 g fiber) 30 min prior to breakfast and lunch lowered body weight (−3.3 kg vs. −1.8 kg, *p* < 0.05) to a greater extent than an isocaloric lower-protein fiber-based shake (1 g protein, 3 g fiber) after 84 days [6]. In addition to satiety effects, protein-containing preloads attenuate post-meal glucose excursions by delaying gastric emptying, slowing glucose absorption, and/or stimulating insulin secretion prior to the main glucose load in the meal [7,8,9]. Oil-containing pre-loads exert similar post-meal effects to protein-containing preloads [10]. Importantly, post-meal glucose levels are the predominate contributor to overall hyperglycemia in individuals without type 2 diabetes. In a cohort of adults without known diabetes (hemoglobin A1c (HbA1c) 5.1–5.5%), post-meal glucose levels contributed to ~81% of overall relative hyperglycemia [11]. Therefore, intake of a fat-, protein-, and fiber-containing preload prior to main meals may be a strategy to promote satiety and reduce postprandial hyperglycemia, which would be expected to promote weight loss and lower the risk of type 2 diabetes.

Substantial evidence shows that nuts are associated with a lower risk of CVD and type 2 diabetes [12]. These findings are supported by randomized controlled trials showing that nuts improve risk factors for CVD [13,14,15] and markers of glycemic control [16,17]. In addition, nuts have high satiety value, and human feeding trials show that nut intake moderates appetite in the post-meal period [18]. Notably, nuts, including peanuts, have been shown to suppress hunger and the desire to eat and increase fullness ratings following intake. However, nuts are energy dense and often excluded from weight loss diets. Evidence to date suggests that nut intake does not promote weight gain in studies targeting weight maintenance [19]. Few studies, however, have evaluated the effect of nut intake in the context of energy-restricted weight loss diets. The aim of this trial was to evaluate the effect of intake of 35 g of peanuts prior to two main meals per day as part of an energy-restricted weight loss diet, compared to a traditional low-fat weight loss diet, on body weight, HbA1c, 2-h glucose, and blood pressure in adults with overweight or obesity at moderate or high risk of type 2 diabetes over 6 months. It was hypothesized that the incorporation of peanuts into a weight loss diet would augment weight loss and improve glycemic control compared to a traditional low-fat weight loss diet.

## 2. Materials and Methods

### 2.1. Study Design

A 6-month 2-arm parallel randomized controlled trial was conducted at the University of South Australia, Adelaide, Australia, to examine the effect of an energy-restricted diet including 70 g/d of peanuts on weight loss, blood pressure, and glycemic outcomes compared with a low-fat weight loss diet. The peanut group was advised to consume 35 g of lightly salted dry-roasted peanuts prior to two main meals per day. Participants in the control group were given education to follow a low-fat diet. Both diet groups were advised to restrict energy intake (women: <5500 kJ/1300 kcal/d; men: <7000 kJ/1700 kcal/d). Participants were randomized at baseline in a one-to-one ratio, using a computer-generated scheme (randomization.com). The study was approved by the University of South Australia Human Research Ethics Committee, and written informed consent was obtained from the participants (Ethics protocol “Longer-term impact of peanuts on body weight and markers of diabetes prevention and control”; Application ID: 203354; Approved 23 October 2020). The study was conducted in accordance with the Declaration of Helsinki.

### 2.2. Participants

Participants were recruited from January 2021 to May 2021 from Adelaide, Australia, using print, social media, and radio advertising. Eligible individuals were >18 years of age, had a body mass index (BMI) >26 kg/m^2^, and were at moderate or high risk of type 2 diabetes (score > 6 points), as assessed by the Australian type 2 diabetes risk assessment tool (AUSDRISK) [20]. In addition, eligible individuals had no health conditions likely to affect the study outcomes and no food allergies/intolerances to peanuts. Exclusion criteria were previous surgery for weight reduction, systolic blood pressure >160 mmHg, currently undergoing medical treatment for acute illness, participation in another ongoing clinical trial, current weight loss diet, and unwillingness to eat peanuts. Individuals taking diabetes or obesity medication were not eligible. Hypertension medication was permitted. Women who were pregnant or planning to become pregnant, or those breastfeeding, were not eligible.

### 2.3. Dietary Intervention

Both the peanut and control group received nutrition education from an accredited practising dietitian to follow an energy-restricted diet. Participants in both groups met with the dietitian monthly throughout the study. Based on previous studies, women and men were counseled to restrict energy intake to 5500 and 7000 kJ, respectively [21,22]. Participants in both groups were asked to keep their exercise patterns constant throughout the study.

Participants in the peanut group were provided education to eat 35 g of peanuts 30 min prior to two of their meals (i.e., 70 g/d) for the entire 6-month study period. Lightly salted dry-roasted peanuts (Fisher Nuts: 1890 kJ/70 g, fat, 35 g/70 g; MUFA, 18.3 g/70 g; sodium, 188 mg/70 g; carbohydrate, 12.5 g/70 g; protein, 17.5 g/70 g) were provided for the duration of the study. Intake of the provided peanuts was assessed by a daily checklist completed by the participants. Participants in the control group were given education to follow a low-fat diet and asked to avoid peanuts and peanut butter for the duration of the study. Dietetic education to follow an energy-restricted diet is reflective of standard care for management of overweight and obesity [23]. Participants in the control group were given a grocery voucher to the same value as the peanuts provided to the peanut group. Participants in both groups were asked to weigh themselves weekly at home between the clinic visits.

### 2.4. Outcomes

Participants attended the research center on 7 occasions (Table 1). At baseline, 3- and 6-months blood samples were taken for measurement of HbA1c, fasting glucose, and insulin, and a 2-h oral glucose tolerance test was performed. Weight was measured monthly throughout the study, and blood pressure was measured every 3 months. Prior to each visit, participants were asked to fast from 12:00 a.m. the night before, with only water permitted. Weight and height were measured in light clothing after removal of shoes, and blood pressure was measured in triplicate, using an automated sphygmomanometer after a 5 min rest. Blood samples were taken at a collection site for an accredited clinical laboratory (Clinpath Pathology, Adelaide) for measurement of HbA1c, fasting glucose, and insulin. Homeostasis model assessment for insulin resistance (HOMA-IR) was calculated according to the following formula: fasting glucose x fasting insulin/22.5 [24]. The 2-h oral glucose tolerance test was performed at the research center. Blood samples were taken in the fasting state and at 120 min following a 75 g glucose drink. Blood samples were analyzed by a commercial laboratory (Clinpath Pathology, Adelaide).

A single non-random 24 h recall was collected at baseline, 3 months, and 6 months, using the Automated Self-Administered 24 h Recall (ASA-24) System (Australia 2016 Version). Completion of a single 24 h recall at each timepoint is recommended for assessing changes in mean usual intake in response to an intervention [25]. Participants were asked to recall intake from midnight to midnight on the day prior. No exclusions were made based on energy intake since all reported energy intakes were deemed plausible. The National Cancer Institute Guidelines for reviewing and cleaning ASA-24 data were followed [26].

### 2.5. Statistical Analyses

Sample size calculations showed that the completion of 50 participants in each group would provide 80% power to detect a 1.7 kg (standard deviation 3.0 kg) difference between the groups (*p* < 0.05) [21]. Weight loss is the primary outcome. All other outcomes are secondary.

All the statistical analyses were performed with SAS (version 9.4; SAS Institute, Cary, NC, USA). All available data from randomized participants were included in data analyses consistent with intent-to-treat principles. Data from participants who withdrew from the study were included when endpoint measures were obtained. The mixed-models procedure does not perform listwise deletion, thus preserving the degrees of freedom; therefore, this analytical approach allowed for inclusion of participants with ≥1 missing data point. The normality of the residuals was assessed by using univariate analysis (PROC UNIVARIATE) to quantitatively evaluate skewness and to visually inspect the distribution and normal probability (Q–Q) plots.

The mixed-models procedure (PROC MIXED) was used to examine the effect of diet on each outcome. Visit was modeled as a repeated effect to account for the repeated-measures design. Diet was modeled as a fixed effect, and the baseline value was included as a covariate. When a main effect of diet, visit, or diet by visit was detected, post hoc pairwise comparisons were conducted and the Tukey–Kramer method was used to adjust for multiple comparisons; data from post hoc testing are presented as the pairwise mean difference and 95% CI with the Tukey–Kramer adjusted *p*-value. Sex effects and sex-by-diet interactions were also evaluated. Statistical significance was set at *p* < 0.05.

## 3. Results

### 3.1. Participants

**Figure 1:**
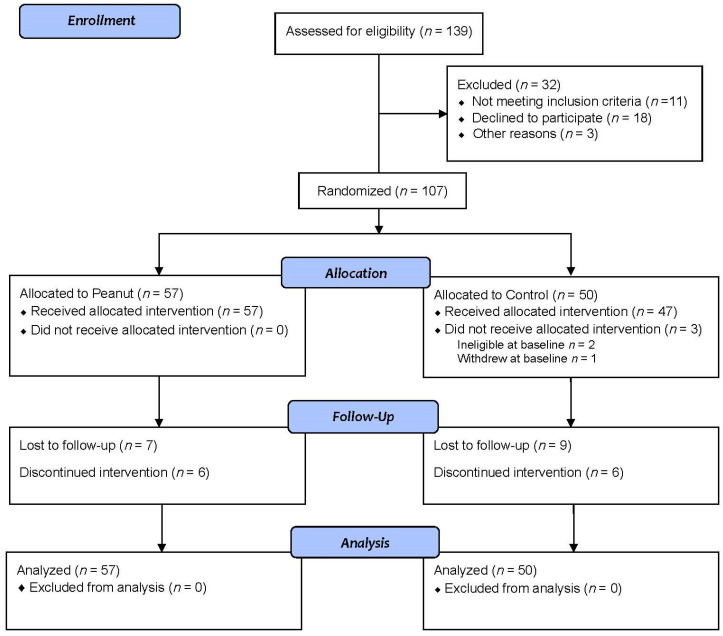
CONSORT flow diagram.

In total, 107 participants were randomized. Of the randomized participants, one withdrew during baseline testing and two were deemed ineligible at baseline. At 3 months, 47 participants randomized to the peanut group and 33 participants randomized to the control group attended the follow-up visit. After 6 months, 44 participants in the peanut group and 32 participants in the control group attended the follow-up visit (Figure 1). At baseline, the two groups were very similar. The cohort has a mean age of 58 years (range 19–79 years), a mean BMI of 33.1 ± 5.4 kg/m^2^, and waist circumference of 109 ± 12.9 cm (Table 2). The peanut group reported that the provided peanuts were consumed on 93% of study days.

### 3.2. Weight

**Figure 2:**
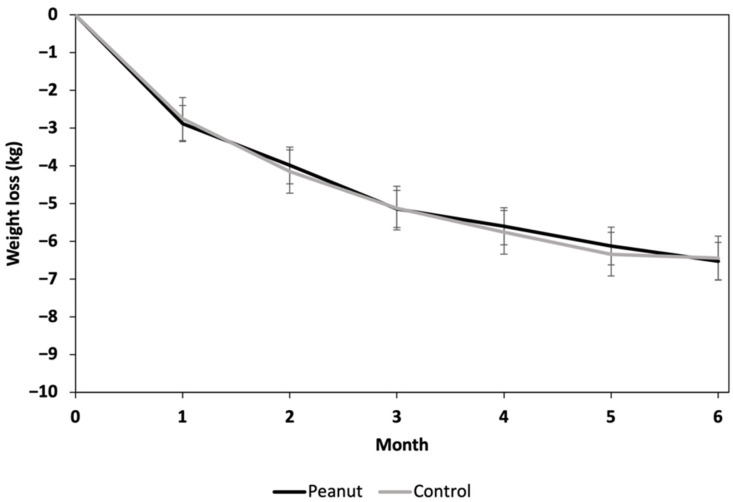
Weight change from baseline in each study group over the 6-month study period. Data presented as least squares means ± standard error of mean. Data were analyzed by using linear mixed models (PROC MIXED; SAS Version 9.4). The effect of diet on change in weight from baseline was examined with visit modeled as a repeated effect and baseline weight included as a covariate.

A visit main effect was observed (*p* < 0.001) for weight; no diet effect (*p* = 0.94) or visit-by-diet interaction (*p* = 0.98) was observed (Figure 2). Compared to the baseline, at 6 months, the peanut group lost 6.72 kg (95% CI, −8.21, −5.23), and the control group lost 6.60 kg (95% CI, −8.35, −4.85); no difference in weight loss was observed between the peanut group and control group at 6 months (mean difference, −0.12; 95% CI, −2.42, 2.18; *p* = 0.92). No sex effects or sex-by-diet interactions were observed. Only three participants in each group did not lose weight at 6 months compared to the baseline.

### 3.3. Blood Pressure

For systolic blood pressure, main effects of diet (*p* = 0.007) and visit (*p* < 0.001) were observed; the diet-by-visit interaction (*p* = 0.063) approached statistical significance (Table 3). Compared to baseline, systolic blood pressure was significantly reduced in the peanut group (−9.46 mmHg, 95% CI, −11.96, −6.95; *p* < 0.001) and the control group (−4.13 mmHg; 95% CI, −7.11, −1.14; *p* = 0.007) after 6 months. The 6-month reduction in systolic blood pressure observed in the peanut group was significantly greater than the corresponding change observed in the control group (between-group mean difference, −5.33 mmHg; 95% CI, −9.23, −1.43; *p* = 0.008). No sex effects or sex-by-diet interactions were observed for systolic blood pressure.

No diet effect or diet-by-visit interaction was observed for diastolic blood pressure. A visit main effect was observed for diastolic blood pressure. Diastolic blood pressure declined in the cohort at 3 (−3.92 mmHg; 95% CI, −5.52, −2.32; *p* < 0.001) and 6 (−4.76 mmHg; 95% CI, −6.40, −3.13; *p* < 0.001) months compared to baseline. No difference in diastolic blood pressure was observed between 3 and 6 months. No sex effects or sex-by-diet interactions were observed.

### 3.4. Glycemic Outcomes

No diet effects or diet-by-visit interactions were observed for fasting glucose, fasting insulin, 2-h glucose, HbA1c, or HOMA-IR (Table 4). Fasting glucose was reduced in the cohort over time (visit *p* < 0.001). Compared to baseline, fasting glucose was lower at 3 months (−0.14 mmol/L; 95% CI, −0.24, −0.04; *p* = 0.004) and 6 months (−0.18 mmol/L; 95% CI, −0.28, −0.08; *p* < 0.001) in the cohort. No sex effect or diet-by-sex interaction was observed for fasting glucose. No main effects of visit, sex, or sex-by-diet were observed for 2-h glucose.

Insulin declined over time in the whole cohort (visit *p* < 0.001). Compared to baseline, insulin was lower at 3 months (−2.62 u/mL; 95% CI, −4.06, −1.19; *p* < 0.01) and 6 months (−3.38 u/mL; 95% CI, −4.85, −1.91; *p* < 0.001). No sex effect or diet-by-sex interaction was observed for insulin. HOMA-IR also declined over time in the whole cohort (*p* < 0.001). Compared to baseline, the HOMA-IR was lower at 3 months (−0.61; 95% CI, −0.93, −0.30; *p* < 0001) and 6 months (−0.84; 95% CI, −1.16, −0.51; *p* < 0.001). No sex effect or diet-by-sex interaction was observed for HOMA-IR.

HbA1c declined in the cohort over time (visit *p* < 0.001). Compared to baseline, HbA1c was lower at 3 months (−0.08%; 95% CI, −0.12, −0.04; *p* < 0.001) and 6 months (−0.13%; 95% CI, −0.17%, −0.09; *p* < 0.001) in the whole cohort. HbA1c was also lower at 6 months compared with 3 months (−0.05%; 95% CI, −0.09, −0.003; *p* = 0.03). A sex effect (*p* = 0.03) was observed, whereby women had higher HbA1c vs. men; however, no diet-by-sex interaction was observed.

### 3.5. Dietary Intake

Main effects of diet, visit, and diet-by-visit were observed for intake of energy, total fat (g and % kJ), MUFA (% kJ), and carbohydrates (% kJ) (Table 5). Post hoc testing showed that energy intake was significantly lower in the control group at 6 months (−1731 kJ; 95% CI, −3231, −231; *p* = 0.01) compared to the peanut group; no between-group difference was observed at 3 months. The percentage of energy from total fat was significantly higher in the peanut group vs. the control group at 3 months (11%; 95% CI, 6, 17; *p* < 0.001) and 6 months (12%; 95% CI, 6, 17; *p* < 0.001). The higher intake of fat in the peanut group was explained by the higher intake of MUFA from the provided peanuts. Compared to the control group, energy intake from MUFA was higher in the peanut group at 3 months (10%; 95% CI, 7, 13; *p* < 0.001) and 6 months (11%; 95% CI, 7, 14; *p* < 0.001). The percentage of energy from carbohydrates was significantly lower in the peanut group vs. the control group at 3 months (−13%; 95% CI, −19, −8; *p* < 0.001) and 6 months (−10%; 95% CI, −16, −5; *p* < 0.001). These data confirm a high compliance level in both groups since the differences reflect intake of a high fat food (i.e., peanuts) vs. the low-fat diet (higher in carbohydrates).

A visit effect was observed for saturated fat, and post hoc testing showed that intake was lower at 3 months compared to baseline in the whole cohort (−1.8%; 95% CI, −3.0, −0.5; *p* = 0.004). A visit effect was also observed for sodium. In the whole cohort, sodium intake was lower at 3 months (−325 mg; 95% CI, −625, −26; *p* = 0.03) and 6 months (−440 mg; 95% CI, −747, −134; *p* = 0.002) compared to the baseline; no diet effect or diet-by-visit interaction was observed for sodium intake. Main effects of diet and diet-by-visit were observed for potassium. Potassium intake was higher in the peanut group vs. the control group throughout the study. Post hoc testing showed no significant difference in potassium intake between the groups at each timepoint (*p* > 0.05 for all). A diet-by-visit interaction was observed for fiber; post hoc testing showed no significant difference in fiber intake between the groups at each timepoint (*p* > 0.05 for all).

## 4. Discussion

This randomized trial showed that a peanut-enriched weight loss diet resulted in similar weight loss to a traditional low-fat weight loss diet. However, greater systolic blood pressure reductions were observed with the peanut-containing weight loss diet vs. the traditional diet at 6 months. Both diets improved fasting glucose and insulin, HOMA-IR, and HbA1c. Collectively, the results of this trial suggest that 70 g/d of peanuts may be included in an energy-restricted weight loss diet without attenuating weight loss over a 6-month period.

Peanuts are energy-dense (24.6 kJ/g or 5.9 kcal/g) and concern has been raised about the potential for habitual intake of nuts to promote weight gain [27]. For this reason, nuts are often avoided by individuals following weight loss diets. In this trial, participants in the peanut group were given 70 g/d (1890 kJ/450 kcal) of peanuts to consume prior to two main meals per day. In the context of dietetic counseling to follow an energy-restricted diet, participants did achieve an energy deficit consistent with clinically significant weight loss (−7.5% of initial body weight) that did not differ from the control group given dietetic counseling to follow an energy-restricted low-fat diet at 6 months. Differences in the macronutrient composition of the diets consumed by each group were observed; however, the differences align with the nutrient profile and servings of peanuts provided. These results are consistent with data showing comparable weight loss with lower-fat diets (<30% of total energy from fat) versus higher-fat diets (>40% of total energy from fat) [5]. However, prior research is less consistent with regard to the effect of lower-fat, higher-carbohydrate diets compared to higher-fat, lower-carbohydrate diets on blood pressure [5].

In the present study, we observed greater systolic blood pressure lowering (−5 mmHg) in the peanut group compared to the control group at 6 months. Based on a recent meta-analysis, a 5 mmHg reduction in systolic blood pressure would be expected to lower the risk of a major cardiovascular event by 10% [28]. In individuals with overweight or obesity, weight-loss is recommended for blood pressure control [29], although the blood-pressure-lowering effect of weight loss is variable across studies [30,31]. A meta-regression of data from randomized controlled trials showed that 1 kg of weight loss lowers systolic blood pressure by 0.36 mmHg [30]. However, an earlier analysis showed that systolic blood pressure was reduced by 1.05 mmHg per 1 kg of weight loss [31]. Since weight loss was comparable between the peanut group and the control group, diet-related differences likely explain the systolic blood pressure lowering observed in the peanut group.

A meta-analysis of 21 randomized controlled trials showed that nut intake lowered systolic blood pressure in individuals without type 2 diabetes (mean difference, −1.29 mmHg; 95% CI, −2.35, −0.22); however, only two of the included studies examined peanuts, and no effect on systolic blood pressure was observed in both studies [32]. A more recent meta-analysis of six randomized controlled trials showed no effect of peanuts on systolic blood pressure [33]. While the results of the present study diverge from prior evidence, it should be noted that relatively few studies have examined the effect of peanuts on blood pressure, and limited studies have evaluated cohorts at high risk of type 2 diabetes undergoing weight loss.

The higher-MUFA/lower-carbohydrate intake in the peanut group may have contributed to the observed systolic blood pressure lowering. A systematic review and meta-analysis of randomized controlled trials including patients with type 2 diabetes showed that the intake of a high MUFA diet lowered systolic blood pressure (WMD, −2.25 mmHg; 95% CI, −3.79, −0.70) compared to a high carbohydrate diet [34]. However, a meta-analysis of 14 randomized controlled trials with no exclusion criteria for the health status of the participants showed that low-saturated-fat, high-MUFA diets did not affect blood pressure compared to low-saturated-fat, high-carbohydrate diets [35]. Thus, the effect of higher MUFA diets on systolic blood pressure remains unclear; however, the dietary source of MUFA may explain some of the inconsistencies. In the meta-analysis by Qian et al., all of the studies included plant-sources of MUFA [34]. Collectively, this evidence suggests that diets high in plant-derived MUFA may have blood-pressure-lowering effects.

In both groups, sodium intake was reduced over the 6-month period, which likely contributed to the systolic blood pressure reductions observed over time. However, the reduction in sodium intake would be expected to lower systolic blood pressure by <1 mmHg based on a meta-regression showing systolic blood pressure lowering of 0.042 mmHg per 1 mmol reduction in sodium excretion per day [36]. Potassium intake was, on average, higher in the peanut group vs. the control group (322 mg), although this increase in potassium intake would only modestly lower systolic blood pressure (<1 mmHg) [36]. Therefore, the changes in sodium and potassium intake likely made a small contribution to the overall systolic blood pressure reductions observed.

In this study, we did not observe any between-group differences in fasting glucose or insulin, HOMA-IR, 2-h glucose, or HbA1c. A meta-analysis of randomized controlled feeding studies showed that replacement of 5% of energy from carbohydrates with MUFA had no effect on fasting glucose, 2-h glucose, or fasting insulin [37]. However, reductions in HbA1c (−0.09%; 95% CI, −0.12, −0.05), 2-h insulin (−20 pmol/L; 95% CI, −32.2, −8.4), and HOMA-IR (−2.4%; 95% CI, −4.6, −0.3) were observed. Therefore, it is possible that replacement of carbohydrate with MUFA has insulin-sensitizing effects that we did not detect in this study because only HOMA-IR was assessed, which primarily reflects hepatic insulin sensitivity. In individuals with impaired fasting glucose, insulin levels are low in the fasting state and insufficient to maintain normoglycemia, which is not accounted for in the HOMA-IR calculation [38].

It is also plausible that the hypothesized improvements in glycemic control were not observed because the MUFA that was consumed as part of the peanut matrix had limited intestinal bioavailability, and therefore did not delay gastric emptying, reduce carbohydrate absorption, and/or stimulate insulin secretion to lower postprandial glucose excursions, which has been observed with the intake of MUFA-rich oil prior to a meal [10]. Given that postprandial glucose levels are a major determinant of overall glycemic control in individuals with impaired glycemic control [11], intake of a more intestinally bioavailable form of peanuts may be needed to attenuate post-meal hyperglycemia to improve overall glycemic control. In a randomized crossover study, it was shown that the addition of 42.5 g of peanut butter to a breakfast meal reduced glucose levels at 15 and 45 min compared to a control breakfast; the meal with 42.5 g of whole peanuts did not affect glucose compared to the control or peanut butter breakfasts [39]. Furthermore, glycemic response to the second meal was significantly lower with the peanut-butter-containing breakfast compared to the control breakfast. Reis et al. also observed lower non-esterified fatty acid (NEFA) levels following the peanut butter meal compared to the control meal [39]. The authors suggest that the peanut-butter-induced improvement in glycemic response was because of increased insulin sensitivity due to the reduced circulating concentration of NEFA; increased fatty acid concentration is known to impair insulin signaling and lead to insulin resistance. Therefore, we may have observed different effects if peanut butter was used instead of whole peanuts. Future studies should investigate whether habitual intake of peanut butter at mealtimes improves longer-term glycemic control.

This study has several strengths, including the randomized controlled design, the 6-month follow-up period, and the provision of nutritional counseling by a dietitian. However, this study is limited by the lack of assessment of 2-h insulin concentration, as well as measurement of insulin sensitivity. Characterization of changes in insulin sensitivity would provide insights into the effect of the diets on reversing insulin resistance and delaying type 2 diabetes. In addition, we did not assess waist circumference or loss of lean and fat free mass following the weight loss diets. The control group in this study was provided with dietetic education to follow an energy-restricted diet, which is reflective of standard care for the management of overweight and obesity. However, since the control group did not consume a preload, no inferences can be made about the superiority of a peanut preload vs. other preloads. Finally, attrition in the control group was greater than in the peanut group, which may have affected our power to detect statistically significant differences in the primary outcome between the groups. However, based on the effect observed and the 95% CI (mean difference, −0.12 kg; 95% CI, −2.42, 2.18) it is unlikely that there was a clinically significant difference between the groups.

## 5. Conclusions

In conclusion, intake of 35 g of lightly salted dry-roasted peanuts prior to two main meals per day, in the context of a weight loss diet, resulted in similar weight loss to a traditional low-fat weight loss diet in adults at high risk for type 2 diabetes after 6 months. No differences in HbA1c, fasting glucose, fasting insulin, or 2-h glucose were observed between the two weight loss diets. Greater reductions in systolic blood pressure were observed with the peanut-containing weight loss diet, which may lower CVD risk.

## Figures and Tables

**Table 1:** Schedule of outcome assessment during the study.

Outcome Assessment	Time (Months)						
	0	1	2	3	4	5	6
Weight	X	X	X	X	X	X	X
Height	X						
Blood pressure	X			X			X
24 h dietary recall	X			X			X
Study food checklist	X	X	X	X	X	X	X
HbA1c	X			X			X
Fasting blood glucose and insulin	X			X			X
2-h glucose tolerance test	X			X			X

HbA1c, hemoglobin A1c.

**Table 2:** Baseline characteristics of all randomized participants.

	Total (*n* = 107)	Peanut (*n* = 57)	Control (*n* = 50)
Age, years	58 ± 14	59 ± 14	58 ± 15
Female, *n* (%)	70 (65)	41 (72)	29 (58)
Weight, kg	92.2 ± 17.2	91.6 ± 17.6	92.9 ± 16.9
BMI, kg/m^2^	33.1 ± 5.4	33.1 ± 4.9	33.0 ± 6.0
Waist circumference, cm	109 ± 12.9	108 ± 13.4	109 ± 12.5
Systolic blood pressure, mmHg	128 ± 16	126 ± 15	129 ± 17
Diastolic blood pressure, mmHg	81 ± 10	81 ± 10	81 ± 10
AUSDRISK Score	15.3 ± 4.7	15.0 ± 4.7	15.6 ± 4.7
Fasting plasma glucose, mmol/L	5.1 ± 0.7	5.1 ± 0.6 ^1^	5.2 ± 0.8 ^2^
Fasting insulin, u/mL	11.1 ± 6.7	10.6 ± 6.9	11.8 ± 6.3 ^2^
HbA1c, %	5.6 ± 0.4	5.6 ± 0.3	5.6 ± 0.6 ^3^
2-h glucose, mmol/L	5.9 ± 2.3	5.7 ± 1.8 ^1^	6.2 ± 2.9 ^2^
Prescribed antihypertensive medication, *n* (%)	14 (13)	5 (9)	9 (18)

Data presented as mean ± standard deviations, unless otherwise stated; ^1^ *n* = 56; ^2^ *n* = 44; ^3^ *n* = 45. AUSDRISK, Australian type 2 diabetes risk assessment tool; BMI, body mass index; HbA1c, hemoglobin A1c.

**Table 3:** The effect of the study diets on blood pressure.

	Peanut Group			Control Group			*p*-Values		
Time (Months)	0 (*n* = 57)	3 (*n* = 47)	6 (*n* = 44)	0 (*n* = 50)	3 (*n* = 33)	6 (*n* = 31)	Diet	Visit	Diet x Visit
SBP, mmHg	127 ± 0.9	119 ± 1.0	117 ± 1.1	127 ± 1.0	122 ± 1.2	122 ± 1.3	0.007	<0.001	0.063
DBP, mmHg	81 ± 0.6	77 ± 0.7	75 ± 0.7	81 ± 0.7	77 ± 0.8	76 ± 0.8	0.52	<0.001	0.70

Data presented as least squares means ± standard error of mean. Data were analyzed by using linear mixed models (PROC MIXED; SAS Version 9.4). The effect of diet on each outcome was examined with visit modeled as a repeated effect and the baseline value included as a covariate. SBP, systolic blood pressure; DBP, diastolic blood pressure.

**Table 4:** The effect of the study outcomes on glycemic outcomes.

	Peanut Group			Control Group			*p*-Values		
Time (Months)	0 (*n* = 57)	3 (*n* = 46)	6 (*n* = 43)	0 (*n*= 44)	3 (*n* = 35)	6 (*n* = 32)	Diet	Visit	Diet x Visit
Fasting glucose, mmol/L	5.12 ± 0.04 ^1^	5.01 ± 0.05	4.99 ± 0.05	5.13 ± 0.05	4.96 ± 0.05 ^4^	4.90 ± 0.06 ^5^	0.37	<0.001	0.46
Fasting insulin, u/mL	10.89 ± 0.52	8.95 ± 0.58	8.14 ± 0.59 ^3^	11.42 ± 0.59	8.15 ± 0.67	7.33 ± 0.70	0.50	<0.001	0.41
2-h glucose, mmol/L	5.84 ± 0.17 ^1^	5.93 ± 0.19 ^2^	6.06 ± 0.19	5.89 ± 0.19	6.30 ± 0.21 ^4^	6.41 ± 0.22 ^5^	0.18	0.09	0.58
HbA1c, %	5.61 ± 0.02	5.50 ± 0.02	5.48 ± 0.02	5.61 ± 0.02 ^2^	5.55 ± 0.02	5.49 ± 0.02	0.21	<0.001	0.32
HOMA-IR	2.49 ± 0.12 ^1^	2.09 ± 0.14	1.88 ± 0.14	2.66 ± 0.14	1.83 ± 0.16 ^4^	1.60 ± 0.17 ^5^	0.35	<0.001	0.17

Data presented as least squares means ± standard error of mean. Data were analyzed by using linear mixed models (PROC MIXED; SAS Version 9.4). The effect of diet on each outcome was examined with visit modeled as a repeated effect and the baseline value included as a covariate; ^1^ *n* = 56; ^2^ *n* = 45; ^3^ *n* = 44; ^4^ *n* = 34; ^5^ *n* = 31. HbA1c, hemoglobin A1c; HOMA-IR, homeostasis model assessment for insulin resistance.

**Table 5:** The effect of the peanut-containing weight loss diet compared to the traditional low-fat weight loss diet on dietary intake assessed by self-administered 24 h recalls.

	Peanut Group			Control Group			*p*-Values		
Time (Months)	0 (*n*= 57)	3 (*n* = 48)	6 (*n* = 44)	0 (*n* = 47)	3 (*n* = 34)	6 (*n*= 32)	Diet	Visit	Diet x Visit
Energy (kJ)	8340 ± 295	7011 ± 322	7657 ± 336	8770 ± 325	6126 ± 382	5926 ± 394	0.01	<0.001	0.005
Protein (g)	91 ± 3.6	87 ± 4.0	97 ± 4.1	91 ± 4.0	72 ± 4.7	79 ± 4.9	0.005	0.02	0.055
Protein (% kJ)	19 ± 0.6	21 ± 0.6	21 ± 0.7	18 ± 0.7	20 ± 0.8	22 ± 0.8	0.52	<0.001	0.27
Total Fat (g)	80 ± 4.0	75 ± 4.4	88 ± 4.6	83 ± 4.4	50 ± 5.2	52 ± 5.4	<0.001	<0.001	<0.001
Total Fat (% kJ)	36 ± 1.1	40 ± 1.2	44 ± 1.2	35 ± 1.2	29 ± 1.4	32 ± 1.4	<0.001	0.03	<0.001
Saturated Fat (g)	29 ± 1.5	21 ± 1.7	24 ± 1.7	30 ± 1.7	18 ± 2.0	18 ± 2.0	0.07	<0.001	0.17
Saturated Fat (% kJ)	13 ± 0.5	11 ± 0.5	12 ± 0.5	12 ± 0.5	10 ± 0.6	11 ± 0.6	0.25	0.005	0.88
MUFA (g)	32 ± 1.9	38 ± 2.1	45 ± 2.1	34 ± 2.1	18 ± 2.4	20 ± 2.5	<0.001	0.09	<0.001
MUFA (% kJ)	14 ± 0.7	21 ± 0.7	23 ± 0.7	14 ± 0.7	11 ± 0.8	12 ± 0.9	<0.001	<0.001	<0.001
PUFA (g)	13 ± 0.7	10 ± 0.8	12 ± 0.8	13 ± 0.8	8 ± 1.0	9 ± 1.0	0.03	<0.001	0.22
PUFA (% kJ)	5.8 ± 0.3	5.1 ± 0.3	5.7 ± 0.3	5.5 ± 0.3	4.8 ± 0.4	5.4 ± 0.4	0.24	0.08	>0.99
Carbohydrates (g)	192 ± 7.5	138 ± 8.1	140 ± 8.5	201 ± 8.2	167 ± 9.7	144 ± 10	0.055	<0.001	0.33
Carbohydrates (% kJ)	39 ± 1.1	33 ± 1.2	30 ± 1.2	39 ± 1.2	46 ± 1.4	40 ± 1.5	<0.001	0.003	<0.001
Total Sugars (g)	81 ± 3.8	66 ± 4.1	66 ± 4.3	86 ± 4.2	70 ± 4.9	70 ± 5.1	0.21	<0.001	0.99
Total Fiber (g)	25 ± 1.2	29 ± 1.3	29 ± 1.3	26 ± 1.3	27 ± 1.5	23 ± 1.6	0.18	0.13	0.03
Sodium (mg)	2319 ± 118	1950 ± 129	1968 ± 135	2380 ± 131	2098 ± 153	1850 ± 158	0.81	0.002	0.61
Potassium (mg)	3287 ± 137	3352 ± 149	3619 ± 156	3442 ± 150	2877 ± 177	2973 ± 183	0.02	0.24	0.02

Data presented as least squares means ± standard error of mean. Data were analyzed by using linear mixed models (PROC MIXED; SAS Version 9.4). The effect of diet on each outcome was examined with visit modeled as a repeated effect and the baseline value included as a covariate. MUFA, monounsaturated fatty acids; PUFA, polyunsaturated fatty acids.

## Data Availability

The data presented in this study are available upon request from the corresponding author.
